# Indirect transfer of pyriproxyfen to European honeybees via an autodissemination approach

**DOI:** 10.1371/journal.pntd.0009824

**Published:** 2021-10-14

**Authors:** Sri Jyosthsna Kancharlapalli, Cameron J. Crabtree, Kaz Surowiec, Scott D. Longing, Corey L. Brelsfoard

**Affiliations:** 1 Texas Tech University, Department of Biological Sciences, Lubbock, Texas, United States of America; 2 Texas Tech University, Center for Biotechnology & Genomics, Lubbock, Texas, United States of America; 3 Texas Tech University, Department of Chemistry, Lubbock, Texas, United States of America; 4 Texas Tech University, Department of Plant and Soil Science, Lubbock, Texas, United States of America; University of California, Davis, UNITED STATES

## Abstract

The frequency of arboviral disease epidemics is increasing and vector control remains the primary mechanism to limit arboviral transmission. Container inhabiting mosquitoes such as *Aedes albopictus* and *Aedes aegypti* are the primary vectors of dengue, chikungunya, and Zika viruses. Current vector control methods for these species are often ineffective, suggesting the need for novel control approaches. A proposed novel approach is autodissemination of insect growth regulators (IGRs). The advantage of autodissemination approaches is small amounts of active ingredients compared to traditional insecticide applications are used to impact mosquito populations. While the direct targeting of cryptic locations via autodissemination seems like a significant advantage over large scale applications of insecticides, this approach could actually affect nontarget organisms by delivering these highly potent long lasting growth inhibitors such as pyriproxyfen (PPF) to the exact locations that other beneficial insects visit, such as a nectar source. Here we tested the hypothesis that PPF treated male *Ae*. *albopictus* will contaminate nectar sources, which results in the indirect transfer of PPF to European honey bees (*Apis mellifera*). We performed bioassays, fluorescent imaging, and mass spectrometry on insect and artificial nectar source materials to examine for intra- and interspecific transfer of PPF. Data suggests there is direct transfer of PPF from *Ae*. *albopictus* PPF treated males and indirect transfer of PPF to *A*. *mellifera* from artificial nectar sources. In addition, we show a reduction in fecundity in *Ae*. *albopictus* and *Drosophila melanogaster* when exposed to sublethal doses of PPF. The observed transfer of PPF to *A*. *mellifera* suggests the need for further investigation of autodissemination approaches in a more field like setting to examine for risks to insect pollinators.

## Author summary

Autodissemination approaches have attracted a significant amount of attention for mosquito control because of the advantages of self-delivery of small amounts of highly potent insect growth regulators (IGRs) such as pyriproxyfen (PPF) to oviposition locations. However, while PPF may be delivered to oviposition locations by the mosquito vehicles, these treated mosquitoes may also be delivering PPF to nectar sources that other insects may visit, in particular important insect pollinators. Here we have examined for the direct transfer of PPF to nectar sources and the indirect transfer to the European honey bee. We show PPF is being deposited on artificial nectar sources and is being indirectly transferred to European honey bees. The results are discussed in reference to the potential risks to important insect pollinators of using autodissemination approaches for mosquito control.

## Introduction

Autodissemination is a method of pesticide self-delivery, which is premised on the use of insects as the delivery agent. This method has recently attracted attention for mosquito control, particularly to target container inhabiting species such as *Aedes aegypti* and *Aedes albopictus*, which transmit dengue, Zika, and chikungunya viruses. Both the mosquitoes and pathogens are spreading globally, and vector control remains the only defense against these diseases [1–4]. Autodissemination is based on the behavior of adult mosquitoes and their attraction to breeding sites, including cryptic sites that mosquito control operators fail to find. There are a couple of advantages to using autodissemination techniques: (1) less labor is required because mosquitoes self-deliver insecticide, and (2) the need for less active ingredient relative to conventional control measures. As currently practiced, autodissemination consists of two methods: (1) adult male mosquitoes can be treated with an Insect Growth Regulator (IGR) and the males deliver lethal doses to breeding containers, which has been dubbed ‘Autodissemination Augmented by Males’ (ADAM) [5, 6] and (2) placing artificial adult resting sites (‘dissemination stations’) in the field, which are attractive to adult mosquitoes, and are treated with a persistent IGR [7]. Upon entering the dissemination station, the adult mosquitoes become contaminated with the IGR. The IGR is lethal to immature mosquitoes when their breeding sites become contaminated by the females that arrive to lay eggs (‘oviposit’) and introduce the IGR. The most commonly used IGR for autodissemination approaches is pyriproxyfen (4-phenoxyphenyl (*RS*)-2-(2-pyridyloxy) propyl ether) (PPF). PPF is a juvenile hormone mimic that disrupts the hormonal system of insects resulting in the arrested development and morphogenesis of the adult stage. PPF has been shown to persist up to four months in the environment and is incredibly potent [8, 9]. The concentration required to prevent mosquito development (LC50) is only 0.012 parts per billion [10].

The intended goal and appeal of autodissemination approaches are that small amounts of highly potent IGRs are delivered to cryptic mosquito breeding sites. Recent field studies suggest that these strategies can be successful at reducing mosquito populations [2, 5, 7, 11, 12]. However, while direct targeting of cryptic locations seems like a significant advantage over large scale applications of insecticides, this could actually be detrimental to nontarget organisms by delivering these highly potent long-lasting growth inhibitors to the exact places that other beneficial insects visit, such as locations where many anthophilous insects seek nectar. PPF is not mosquito specific and has been shown to inhibit the development of other insects [13–15] and to impact the physiology of non-target invertebrates [16, 17]. The effects of PPF on pollinators such as honey bees (*Apis mellifera*) are of particular concern [18–20]. We know that male and female mosquitoes seek out nectar sources, and if exposed to a growth inhibitor they could be delivering lethal doses where important pollinators such as honey bees can be contaminated with PPF. PPF has previously been shown to impact adult and immature honey bees if brought back to the colony and could be a potential contributing factor to colony collapse [18, 21]. Several studies have examined the effects of PPF on *A*. *mellifera* related to the application of agricultural and mosquito control formulations that are typically applied to vegetation. Mortality in *A*. *mellifera* adults was previously observed after exposure to PPF through feeding, topical, or contaminated substrate [22]. Exposure to NyGuard (10% Pyriproxyfen) was shown to negatively affect the survival of *A*. *mellifera* foragers and the metabolism and egg development of the silkworm *Bombyx mori* [18, 23]. Previous work has also demonstrated similar reductions in fecundity in *Ae*. *albopictus*, *Ae*. *aegypti*, *Anopheles gambiae*, and *Anopheles arabiensis* after PPF exposure [24–27].

Here we performed a series of studies examining the dissemination of PPF via *Ae*. *albopictus* males to the European honeybee, *A*. *mellifera*, an important non-target and pollinator species. Results suggest the direct and indirect transfer of PPF to artificial nectar sources from *Ae*. *albopictus* males. Furthermore, *A*. *mellifera* when in the same cages with PPF treated *Ae*. *albopictus* were also contaminated with PPF. The results suggest a potential risk of using autodissemination approaches to non-target species, particularly important insect pollinators. We discuss the results in the context of the use of autodissemination techniques for mosquito control.

## Material and methods

### *Drosophila* rearing, PPF application, and fecundity assays

*Drosophila melanogaster* was chosen as a model organism to examine for non-target effects of PPF because of the availability of well-established rearing protocols and known biology. To examine for effects on fecundity, *D*. *melanogaster* (wild-type Oregon-R with hsY) were reared in 31.75 mm x 102 mm culture vials with formula 4–24 Instant *Drosophila* media at room temperature (Carolina Scientific, Burlington, NC, USA). Media was changed monthly by anesthetizing flies with chloroform and transferring to a new vial with fresh media. Eggs were collected on apple juice agar plates. Apple juice agar plates were prepared using 4.5g of agar powder (Alfa Aesar, Haverhill, MA, USA) mixed with 150mL of distilled water that was dissolved on a hot plate at 150°C. In a separate flask, 5g of sucrose was mixed in with 50mL of apple juice and heated until the sucrose was dissolved. The solutions were then combined and 5mL of 20% Methyl 4-hydroxybenzoate (dissolved in 100% ethanol) was added to prevent fungal growth. The final solution was then poured into 50mL clear plastic soufflé cups (Pactiv, Lake Forest, IL, USA), with approximately enough solution so the bottom of the cup was covered in the medium, and allowed to solidify at room temperature. A small quantity of yeast paste was then placed onto the center of an apple-agar to encourage egg-laying.

Twenty-five mated 24–48 hr old *D*. *melanogaster* females were anesthetized using chloroform and placed in cardboard mailing tubes (63.5 mm diameter and 20.3 cm long), capped on both ends with No-See-Um netting (Equinox, Williamsport, PA, USA). Flies were allowed to wake up and then were treated with a 1:1 mixture of Esteem 35 WP (Valent Biosciences, Libertyville, IL, USA) (35% pyriproxyfen) and fluorescent powder (Yu Mingjie pigments, Longdong, Shenzhen, China) using handheld bellow duster (Harris Manufacturing Co. LLC, Cartersville, NC, USA). Treated flies were then placed in a 140 mL Soufflé cup (Pactiv, Lake Forest, IL, USA) with an apple juice agar plate enclosure covered with No-See-Um netting. As a control, 25 *D*. *melanogaster* females were also left untreated. After 48 hours, eggs were counted on the surface of the apple juice agar media using a stereomicroscope. Each treatment consisted of four replicates.

### Mosquito rearing, PPF application, and fecundity assays

*Ae*. *albopictus* used for lab cage and bioassay experiments were from a colony started from eggs collected in Lubbock, TX, USA, and reared for ten generations. Mosquitoes were maintained at 28 ± 2°C and 80 ± 5% relative humidity, and a 16:8 light-dark cycle. Larvae were fed a 60g/L bovine liver powder (MP Biomedicals, Santa Ana, CA, USA) slurry ad libitum. Adults were also provided a 10% sucrose solution. Adult females were fed bovine blood using an artificial blood feeder. Eggs were collected in 140 mL Souffle cups (Pactiv, Lake Forest, IL, USA) containing 100 mL deionized water lined with seed germination paper (Anchor paper company, St. Paul, MN, USA). All eggs were allowed to mature for ~5 days prior to hatching. Eggs were hatched in a 1:1 mixture of fermented: DI water.

To examine for sublethal effects of PPF exposure, fifteen female *Ae*. *albopictus* 24–36 hrs post-emergence were treated with a 1:1 mixture of Esteem 35 WP (Valent Biosciences, Libertyville, IL, USA) (35% pyriproxyfen) and fluorescent powder (Yu Mingjie pigments, Longdong, Shenzhen, China) using handheld bellow duster (Harris Manufacturing Co. LLC, Cartersville, NC, USA) in a cardboard mailing tube (63.5 mm diameter and 20.3 cm long), capped on both ends with No-See-Um netting (Equinox, Williamsport, PA, USA). As a control, fifteen females were left untreated. Treated and untreated females were introduced into 24.5 cm x 24.5 cm x 24.5 cm BugDorm-4S2222 insect rearing cages (MegaView Science Co., Taichung, Taiwan) with fifteen untreated males. A 140 mL Souffle cup (Pactiv, Lake Forest, IL, USA) containing 100 mL deionized water lined with seed germination paper (Anchor paper company, St. Paul, MN, USA) was placed in each cage as an oviposition container. Females were fed bovine blood using an artificial blood feeder and sausage casing membrane. Egg papers were collected five days post the addition of males and the eggs were counted using a stereomicroscope. Each treatment consisted of five replicates.

### Assembly and testing for PPF dissemination to artificial nectar sources

**Fig 1:**
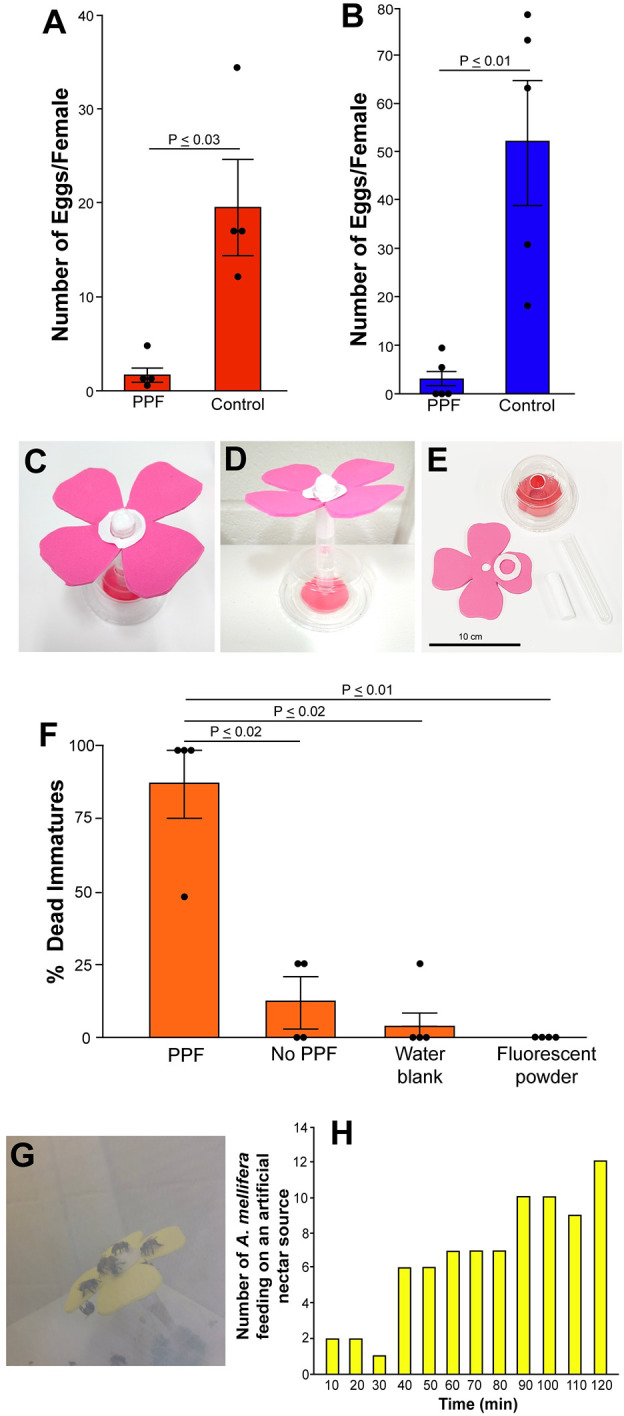
(A) Examination for effects on the fecundity of *D*. *melanogaster* females when exposed to PPF. Bars represent the mean ± SEM number of eggs oviposited by PPF treated (N = 4) and untreated females (N = 4). The horizontal line connecting bars represent significant differences using a pairwise Wilcoxon test, P = 0.02. (B) Examination of the reduction of the fecundity of *Ae*. *albopictus* females when exposed to PPF. Bars represent the mean ± SEM number of eggs oviposited by PPF treated (N = 5) and untreated (N = 5) females. The horizontal line connecting bars represent significant differences using a pairwise Wilcoxon test, P = 0.008. (C-E) Artificial nectar source components and setup. (F) Examination for non-specific transfer of PPF from PPF treated *Ae*. *albopictus* males to artificial nectar sources using the cotton wick from the artificial nectar source in bioassays. Horizontal line connecting bars represent significant differences between treatments using pairwise Wilcoxon tests, P ≤ 0.02, Bonferroni corrected. (G) Image of *A*. *mellifera* acquiring sucrose from an artificial nectar source in laboratory cages. (H) The number of *A*. *mellifera* feeding on the artificial nectar source in laboratory cages determined by counts conducted every ten minutes.

Artificial nectar sources were constructed as a way to examine for PPF transfer from treated mosquitoes. Artificial nectar assemblies were made with a 12 x 75 mm 5 mL culture tube (VWR, Radnor, PA, USA) filled with ~4 mL 25% sucrose solution and a #2 medium sterile cotton dental wick and a floral pattern cut out of Creatolgy art foam (Michaels Inc., Irving, TX, USA) along with ~1.5 cm Whatman (Little Chalfont, Buckinghamshire, UK) 410 filter paper ring that surrounded the cotton dental wick. The artificial floral structure was then placed in a 50 mL soufflé cup with modeling clay as a weight to keep the nectar source upright (Fig 1C, 1D, and 1E). To investigate whether *Ae*. *albopictus* would contaminate artificial nectar sources, fifteen PPF-fluorescent powder mixture treated males or untreated males and fifteen untreated females were placed in four replicate 24.5 cm x 24.5 cm x 24.5 cm BugDorm-4S2222 insect rearing cages (MegaView Science Co., Taichung, Taiwan) with an artificial nectar source. After four days, the cotton wick from the nectar source was removed and examined for PPF transfer from males using bioassays. Bioassays were conducted in 20 mL glass scintillation vials (Cole-Parmer, Vernon Hills, IL, USA) filled with 10 mL distilled water and two drops of 60g/L liver powder slurry (MP biomedicals, Santa Ana, CA, USA). Four third instar *Ae*. *Albopictus* larvae were added to each vial. As a negative control, water blank bioassays with only DI water were conducted with mosquito larvae. To also test for the effects of fluorescent powder, bioassays were setup with 5 ng/mL of fluorescent powder. Bioassays were monitored for 12 days, and mosquitoes were scored as either dead larvae or pupae, or an emerged adult.

To determine if *A*. *mellifera* would visit an artificial nectar source, workers were collected from colonies maintained at the Texas Tech University Quaker Avenue Research Farm in Lubbock, TX, USA (33°36’03” N 101°54’28” W). *A*. *mellifera* were collected approximately 30 min prior to being released into laboratory cages. To limit contact with individual bees, a hand-held DC vacuum (BioQuip Products, Compton, CA USA) was used to collect approximately 30–50 foraging bees at hive entrances and immediately transport them to the laboratory prior to each experiment. To examine for foraging behavior of *A*. *mellifera* in laboratory cages, 15 workers were placed in a 24.5 cm x 24.5 cm x 24.5 cm BugDorm-4S2222 insect rearing cage (MegaView Science Co., Taichung, Taiwan) containing an artificial nectar source. The total number of *A*. *mellifera* acquiring sucrose on the artificial nectar source was recorded every 10 min for approximately two hours.

### Non-target laboratory cage assays

**Fig 2:**
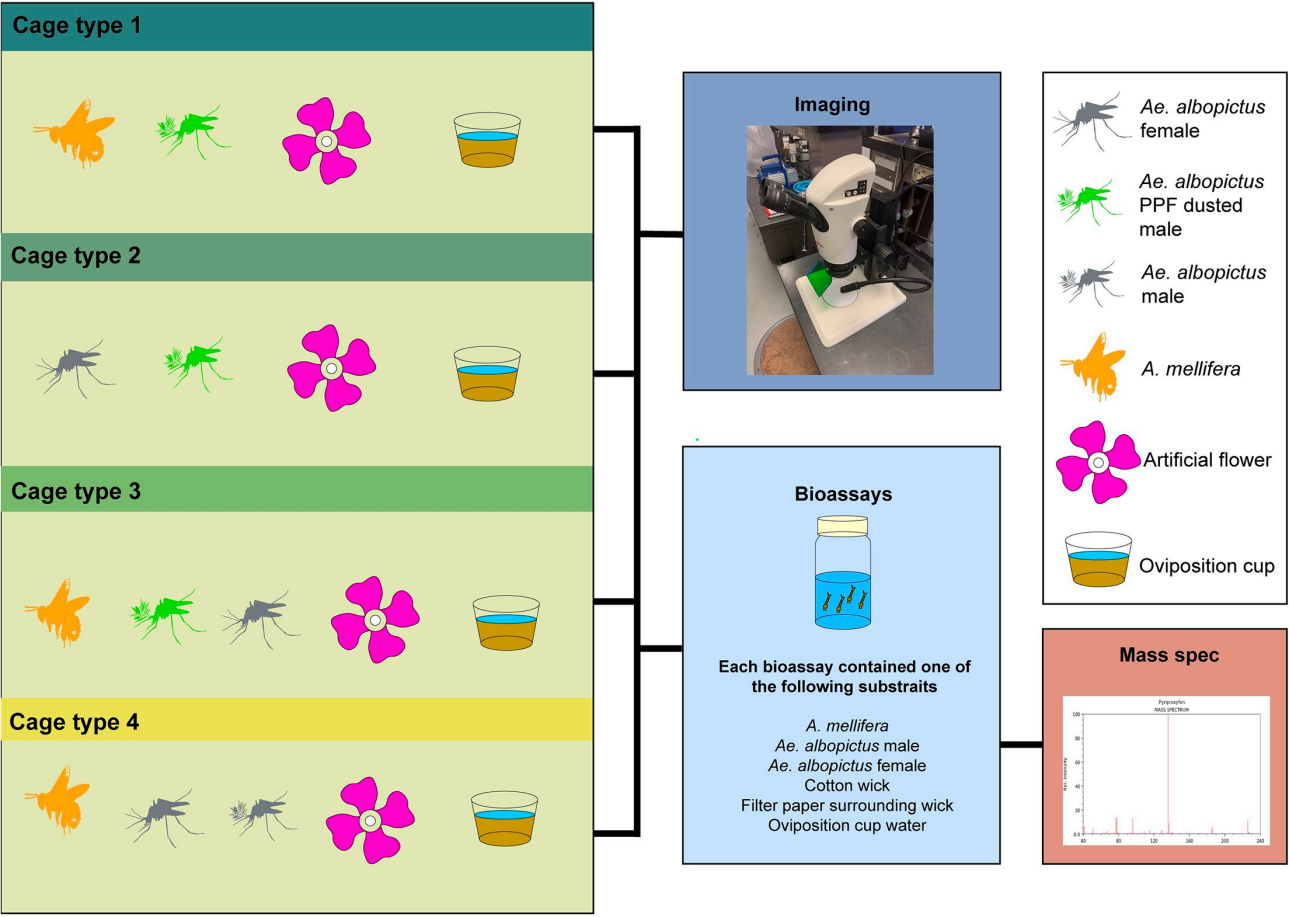
Insects and materials added to each cage type, and the workflow for examination of the transfer of PPF-fluorescent powder mixture to nectar sources, insects, and oviposition cups using imaging, bioassays, and mass spectrometry analysis.

To examine for PPF transfer to non-target *A*. *mellifera* and nectar sources four cage types were set up. Each cage type contained a different combination of 15 treated or 15 untreated *Ae*. *albopictus* males, 15 *Ae*. *albopictus* females, and eight *A*. *mellifera* workers, which are outlined in Fig 2. Male and female mosquitoes and bee mortality were recorded every 24 hours. After five days, the trial was terminated by closing each cage type. Water from the oviposition cup, filter paper, cotton wick, *A*. *mellifera*, male and female *Ae*. *albopictus* were collected and stored at -20°C for later use in bioassays and for mass spectrometry analysis. All experimental treatments were replicated four times. Females were not offered a blood meal over the five-day period.

To confirm the presence of the PPF-fluorescent powder mixture on nectar source materials and insects, images were taken using Leica S9i stereo microscope with an integrated camera, UV light adapter, and GO green only filter (NIGHTSEA, Lexington, MA, USA) at 10-20X magnification. As additional confirmation of the presence of PPF, bioassays were conducted as described previously. *Ae*. *albopictus* treated or untreated males and females, oviposition cup water, the uppermost portion of the sucrose wick (~5 mm), filter paper surrounding the wick, and bees were added to individual bioassays. Bioassays were monitored for immature mortality daily until all immatures were deceased or adults emerged. At least two technical replicate bioassays were completed for each material or insect collection type except for the cotton wick and filter paper because there was only one of these in each of the four replicate cages outlined in Fig 2. Mass spectrometry was also used to detect and quantify the amount of PPF that was found on artificial nectar sources, *A*. *mellifera*, and *Ae*. *albopictus* females and males. PPF was washed off from the materials using 1.0 mL of methanol (Millipore Sigma, Burlington, MA, USA, - 99.9% HPLC grade) in 2.0 mL Eppendorf tubes. The materials and methanol in tubes were vortexed for 4 min at 25°C. The tube was then centrifuged at 16000 RCF for 5 min in a microcentrifuge. 750 μl of the supernatant was then transferred to 2 mL colored glass vials and used for detection of PPF using liquid chromatography-mass spectrometry (LC-MS). To detect PPF in oviposition cup samples 30 mL of water from oviposition cups was extracted in 12 mL of chloroform (Fisher scientific, Walthan, MA, USA—98% HPLC grade). The organic layer, where PPF was expected to dissolve was concentrated by evaporating it in a fume hood for ~72 hours. The residue was dissolved into 750 μl of methanol (VWR BDH chemicals, Radnor, PA, USA—99.8%). An Ultimate 3000 HPLC system with TSQ Vantage triple Quadrupole Mass Spectrometer (Thermo Scientific, Waltham, MA, USA) was used to quantify the amount of PPF. The separation was performed on Scherzo SM-C18 Column 150x2mm, 3μm, (Imtakt, Portland, OR, USA) using a mobile phase containing 10mM Ammonium acetate, 10% Acetonitrile and 0.05% Formic acid at a flow rate of 200μL/min. TSQ Vantage Triple Quadrupole Mass Spectrometer was used with Electrospray Ionization using positive ion SRM with parent ion of 322 m/z and product ion of 96 m/z. An electrospray voltage of 3kV was used and the collision energy was 10V. Calibration was performed using PPF standard solutions in methanol in the range of 0 ng/mL to 17 ng/mL. The detection limit using the described methodology was 0.001 ng/mL, and any number below this limit was considered a negative test for PPF.

### Statistical analyses

Differences in the number of eggs produced by PPF treated and untreated *D*. *melanogaster* and *Ae*. *albopictus* were determined using Kruskal-Wallis and Pair-wise Wilcoxon tests. The number of dead immatures in bioassays examining for dissemination of PPF from treated male *Ae*. *albopictus* to the artificial nectar source were compared by performing an arcsine square root transformation on the proportion dead immatures and Pair-wise Wilcoxon tests. Non-parametric tests were used because the data did not meet the assumptions of parametric statistics (i.e., tests for normality and equality of variance). The number of dead immatures in bioassays from non-target cage assays was compared by performing an arcsine square root transformation of the proportion of dead immatures and an ANOVA. Transformed immature bioassay data were checked for deviations for normality and equality of variance. Pairwise comparisons were performed using t-tests. The survivorship of treated and untreated male and female *Ae*. *albopictus* and *A*. *mellifera* were compared using a log-rank survivorship analysis. All statistical tests were performed using JMP vs. 16 (SAS, Cary, NC, USA). All experimental data are provided in S1–S6 Data.

## Results

### Examination for sublethal effects of PPF on *D*. *melanogaster* and *Ae*. *albopictus*, PPF dissemination to artificial nectar sources, and *A*. *mellifera* artificial nectar source feeding

Exposure to 0.001 PPM PPF was shown to significantly reduce the fecundity of *D*. *melanogaster* (Pairwise Wilcoxon, Chi-sq = 5.4, DF = 1, P = 0.02), and *Ae*. *albopictus* females (Pairwise Wilcoxon, Chi-sq = 7.0, DF = 1, P = 0.008) (Fig 1A and 1B). *Ae*. *albopictus* males were demonstrated to deposit PPF on artificial nectar sources (Fig 1C, 1D, and 1E) in laboratory cages by placing PPF treated males in cages with an artificial nectar source and examining for PPF using bioassays. Bioassays with cotton wicks from treatment cages showed a significant reduction in larval survivorship (Kruskal-Wallis, Chi-sq = 11.4, DF = 3, P ≤ 0.01), suggesting cross-contamination of the nectar source by PPF treated *Ae*. *albopictus* males (Fig 1F). As a control, we also performed bioassays to determine if any lethal effects were the result of the fluorescent powder used in the dust mixture. No lethality was observed in these bioassays suggesting that the fluorescent powder did not affect larval survivorship (Pairwise Wilcoxon, P ≤ 0.01) (Fig 1F).

*A*. *mellifera* placed in laboratory cages were also observed to forage on the artificial nectar sources. After acclimation to the laboratory cage conditions over a 2 h period, 12 of 15 bees were observed on the artificial nectar source (Fig 1G and 1H).

### Evidence of PPF transfer to non-targets and nectar sources in laboratory cages using imaging, bioassays, and mass spectrometry

**Fig 3:**
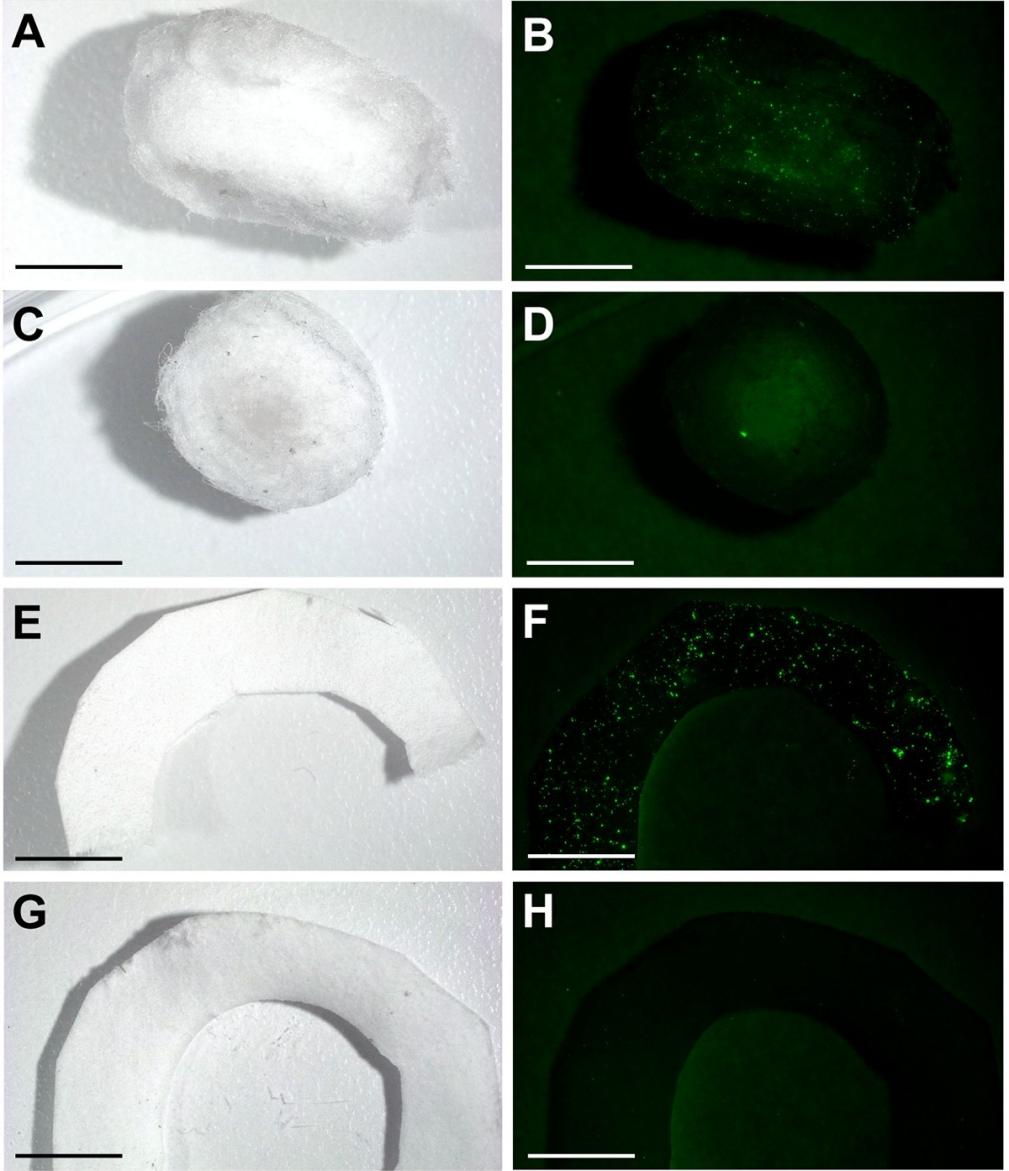
Image of cotton wick from a cage with PPF-fluorescent powder mixture treated *Ae*. *albopictus* males (A) under visible and (B) UV light. Image of cotton wick from a cage with untreated *Ae*. *albopictus* males (C) under visible and (D) UV light. Image of the filter paper ring surrounding the cotton wick from a cage with PPF treated *Ae*. *albopictus* males (E) under visible and (F) UV light. Image of the filter paper ring surrounding the cotton wick from a cage with untreated *Ae*. *albopictus* males (G) under visible and (H) UV light. The scale bars represent 5 mm.

**Fig 4:**
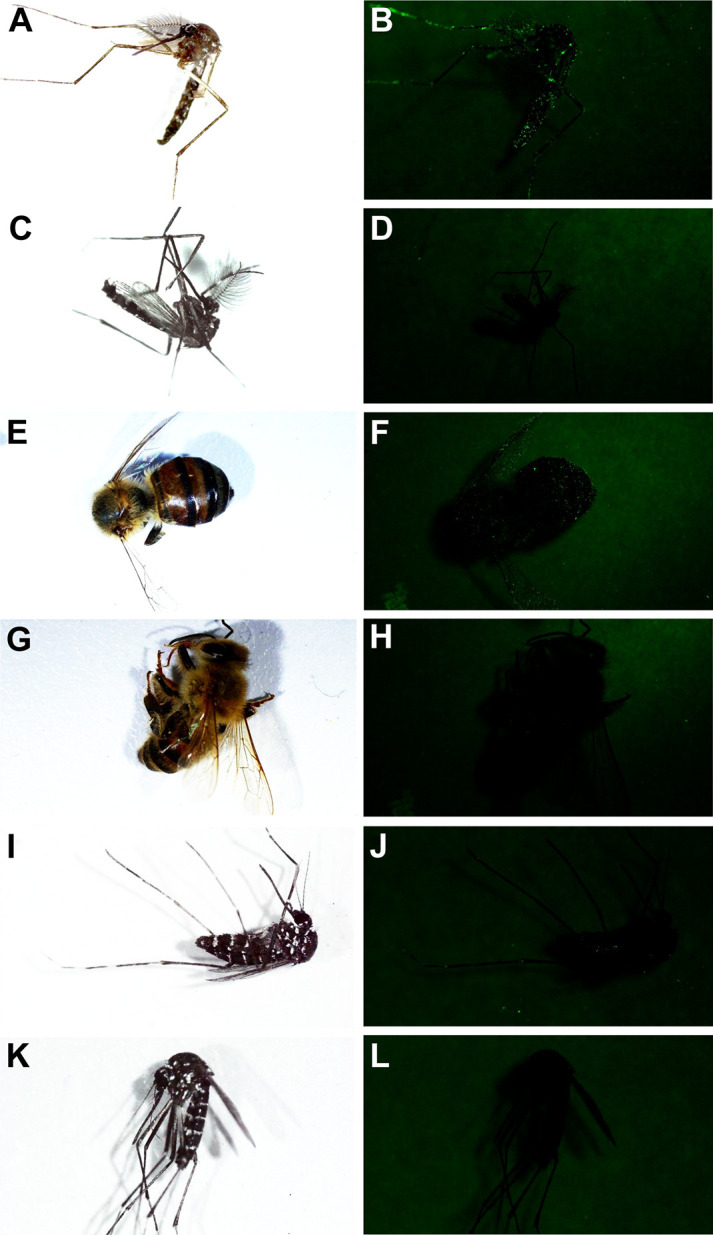
Images of an *Ae*. *albopictus* PPF-fluorescent powder mixture treated (A and B) and untreated (C and D) male collected from a laboratory cage under visible and UV light, showing the presence and absence of PPF-fluorescent powder after males had been in cages for five days, respectively. Images of *A*. *mellifera* collected from (E and F) cages with PPF treated *Ae*. *albopictus* males and (G and H) from cages with untreated *Ae*. *albopictus* males demonstrating the transfer of PPF-fluorescent powder mixture to *A*. *mellifera* in the presence of treated males. Images of *Ae*. *albopictus* females (I and J) collected from cages with treated *Ae*. *albopictus* males and (K and L) from cages with untreated *Ae*. *albopictus* males demonstrating the transfer of PPF-fluorescent powder mixture to con-specific females in the presence of PPF treated males.

Images of artificial nectar source materials collected from cage types 1, 2, and 3 show there is transfer of the PPF-fluorescent powder mixture to the cotton wick (Fig 3) and filter paper ring surrounding the cotton wick on the artificial nectar sources (Fig 3). PPF transfer to *Ae*. *albopictus* females was also observed in cage type 2 and 3 as suggested by the presence of fluorescent powder on untreated females placed in the cages (Fig 4I and 4J). Images of *A*. *mellifera* collected from cage type 3 show the indirect transfer of PPF from treated *Ae*. *albopictus* males and artificial nectar sources (Fig 4A, 4B, 4E and 4F). In contrast, no fluorescence was observed on insects or artificial nectar source materials collected in cage type 4 containing untreated *Ae*. *albopictus* males (Fig 3, Fig 4C, 4D, 4G, 4H, 4K and 4L). The proportion of artificial nectar source materials, mosquitoes, and *A*. *mellifera* in each cage type that had evidence of the PPF-fluorescent powder mixture are shown in S1 Table.

**Fig 5:**
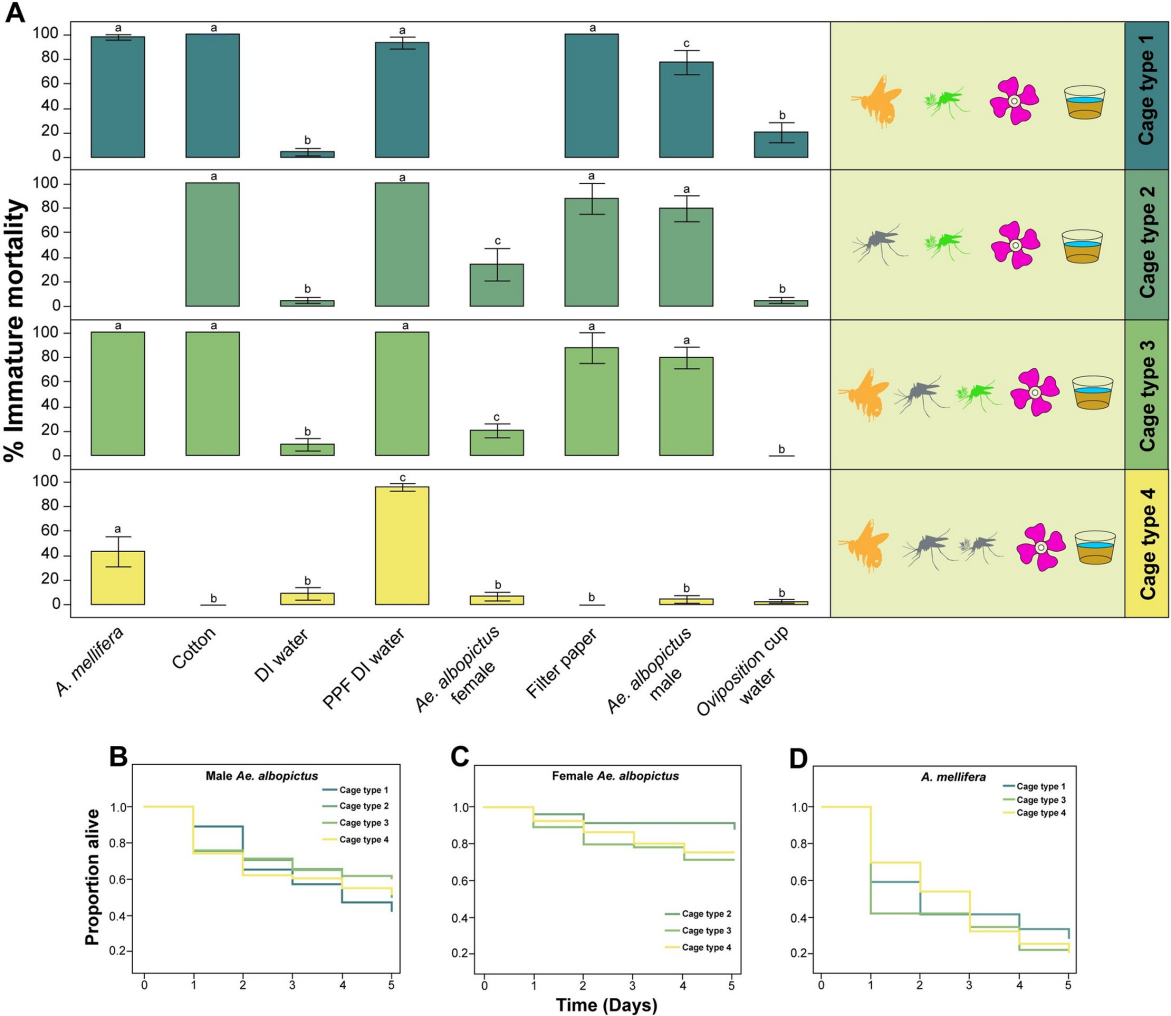
(A) Immature *Ae*. *albopictus* mortality in bioassays conducted using insects and materials collected from cage types 1–4. All data is represented by the mean immature mortality ± SEM. Letters above each bar represent significant differences as determined by t-tests, P < 0.05, (B) Survival plots of male *Ae*. *albopictus* (N = 4), (C) female *Ae*. *albopictus* (N = 4), and (D) *A*. *mellifera* (N = 3). All survival plots are mean numbers of surviving insects on each day for each cage type.

To examine for dissemination of PPF from treated males directly to nectar sources and oviposition containers, and indirectly to female *Ae*. *albopictus* and *A*. *mellifera*, we performed a series of bioassays on insects, oviposition cup water, and artificial nectar source materials from the four cage types with a different combination of PPF treated and untreated *Ae*. *albopictus* and *A*. *mellifera* (Fig 5A). A significant lethal effect was observed in bioassays containing materials from an artificial nectar source, *A*. *mellifera*, and PPF treated *Ae*. *albopictus* males collected from cage type 1 (ANOVA, F = 34.4, DF = 6, P < 0.0001) (Fig 5A). To examine for the dissemination of PPF from treated males to nectar sources and oviposition containers without the presence of *A*. *mellifera*, cage type two consisted of only PPF treated *Ae*. *albopictus* males and untreated females (Fig 5A). A significant lethal effect was observed in bioassays with materials and insects collected from cage type 2 (ANOVA, F = 22.0, DF = 6, P < 0.0001) (Fig 5A). To examine for direct and indirect PPF transfer, cage type 3 consisted of PFF treated males, untreated females, *A*. *mellifera*, and an artificial nectar source. A significant lethal effect was observed in bioassays with materials and insects in the presence of female *Ae*. *albopictus* mosquitoes (ANOVA, F = 70.0, DF = 7, P < 0.0001) (Fig 5A). Lastly, cage type 4 consisted of untreated *Ae*. *albopictus* males and females, *A*. *mellifera*, and an artificial nectar source. A low level of mortality was observed in bioassays containing *A*. *mellifera* and a high level of mortality in bioassays directly inoculated with PPF (ANOVA, F = 24.9, DF = 7, P < 0.0001) (Fig 5A).

No difference in male *Ae*. *albopictus* survivorship was observed when comparing PPF treated to untreated individuals in the four cage types (Log-rank, Chi-square = 2.42, DF = 3, P = 0.49) (Fig 5B). Greater than 70% of females were observed to be alive on day five when cages were closed. A difference was observed in the survivorship in comparisons of females in cage types 2, 3, and 4 (Log-rank, Chi-square = 7.87, DF = 3, P = 0.02), wherein 88% in Cage type 2 were observed to be alive on day five compared to 76% and 73% in Cage type 3 and four respectively (Fig 5C). No difference in *A*. *mellifera* survivorship was observed in cage types 1, 3, and 4 (Log-rank, Chi-square = 0.59, DF = 2, P = 0.75). *A*. *mellifera* mortality was ~25–30% in all cage types at day five (Fig 5D).

**Fig 6:**
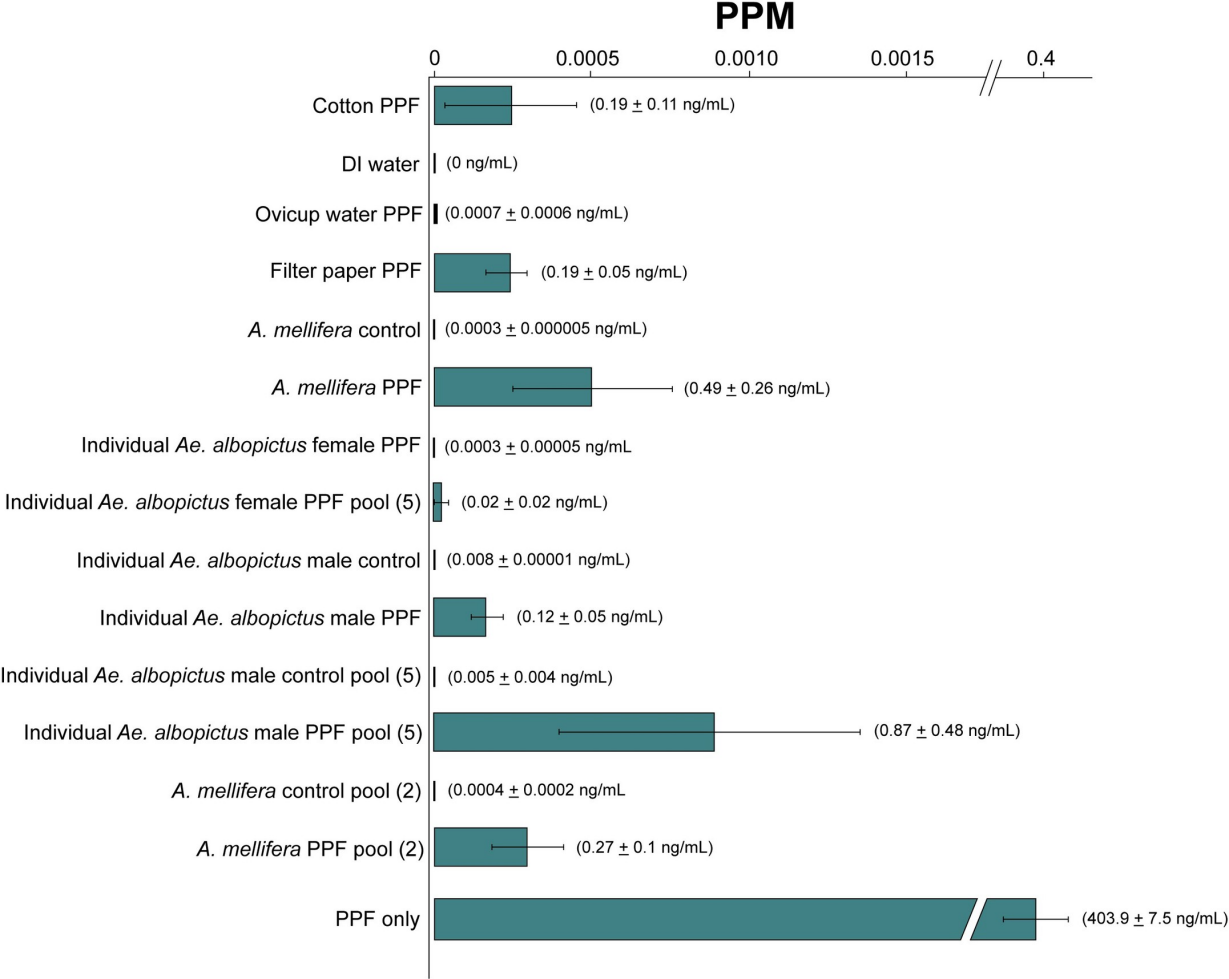
Mass spectrometry HPLC PPF quantification from *Ae*. *albopictus*, *A*. *mellifera*, and materials collected from cages with PPF treated and untreated males (Cage types 3 & 4). All values represent the mean ± SEM of PPF in parts per million (PPM). The mean ± SEM of concentrations of PPF are also listed ng/mL to the right of each bar.

To quantify the amount of PPF on individual and pooled *Ae*. *albopictus* and *A*. *mellifera*, and artificial nectar sources, we performed liquid-chromatography-mass spectrometry (LC-MS) analyses. Insects and nectar source materials and oviposition water were collected from cage types 3 and 4. Results suggest the presence of PPF on *A*. *mellifera*, *Ae*. *albopictus* females, and artificial nectar source materials from cages where PPF treated males were released (Fig 6). The most abundant amounts of PPF were observed on PPF treated *Ae*. *albopictus* males (0.12 ± 0.05 ng/mL) and A. *mellifera* workers (0.49 ± 0.26 ng/mL) (Fig 6). A mean concentration of PPF 0.19 ± 0.11 ng/mL and 0.19 ± 0.05 ng/mL was also observed on the cotton wick and filter paper of the artificial nectar source, respectively (Fig 6).

## Discussion

Here we performed assays to understand the potential for non-target effects of the ADAM approach on insect pollinators. Results suggest *A*. *mellifera* can be contaminated with PPF indirectly through the visitation of artificial nectar sources in the presence of PPF treated male *Ae*. *albopictus* mosquitoes. Studies were performed in laboratory cages and conditions could have impacted *A*. *mellifera* worker foraging behavior; however, bees were observed to forage on the artificial nectar sources for up to five days, suggesting our findings expose a potential route of indirect transfer of PPF to *A*. *mellifera* (Fig 1G and 1H). In addition, data suggest PPF treated *Ae*. *albopictus* males transferred PPF to *Ae*. *albopictus* female mosquitoes, artificial nectar source materials, and oviposition sites. However, only minor amounts of PPF were transferred to oviposition sites (Fig 6 and S6 Data). This observed result may be due to the females not being blood-fed in laboratory cages and therefore they were not attracted to oviposition sites for egg deposition, resulting in little if any transfer of PPF. Furthermore, the presence or absence of *Ae*. *albopictus* females and/or *A*. *mellifera* in laboratory cages did not impact the rate of direct PPF transfer to nectar sources and indirect transfer to *A*. *mellifera* workers.

Mass spectrometry data and bioassays confirm the presence of PFF on *A*. *mellifera* from cages with PPF treated *Ae*. *albopictus* males (Fig 6 and S6 Data). Fluorescent imaging shows bees have PPF-fluorescent powder mixture on their cuticle from indirect transfer from nectar sources (Fig 4 and S1 Table). It is also interesting to note that *A*. *mellifera* had significantly higher amounts of PPF according to mass spectrometry analyses compared to PPF treated *Ae*. *albopictus* males and the cotton wick and filter paper components of the artificial nectar source (Fig 6). This suggests a chain of transfer from PPF treated males to the artificial nectar source to *A*. *mellifera* individuals and perhaps back to male and female mosquitoes that visit the same nectar source. Grooming behavior is assumed to play a role in how PPF accumulated on the abdomen and thorax of *A*. *mellifera* after visiting the artificial nectar source. Furthermore, the anatomical features of bees and the larger surface area of their legs and abdomen could have resulted in a greater accumulation of PPF on their bodies when visiting contaminated nectar sources. We speculate that *A*. *mellifera* after visiting PPF contaminated nectar sources in a wild setting would then deliver PPF back to their hive where contact and communication with nestmates would spread the PPF to other members of the colony, and potentially impact immature bees. Furthermore, *A*. *mellifera* will travel longer distances to forage compared to *Ae*. *albopictus*, so the potential for vectoring PPF across landscapes is high with the observed indirect transfer from nectar sources. Previous studies have demonstrated sublethal and lethal effects of pyriproxyfen on bee colonies [18, 20]. We have also demonstrated PPF exposure can reduce the fecundity of *D*. *melanogaster* and *Ae*. *albopictus* females. While both species are not considered pollinators, here we have used them as model organisms to show the possibility of unintended fecundity effects to insects due to PPF exposure via autodissemination approaches. Similar reductions in fecundity or other fitness effects could occur in other insect pollinators species when exposed to PPF. Previous work has also demonstrated similar effects in *Ae*. *albopictus*, *Ae*. *aegypti*, *An*. *gambiae*, and *An*. *arabiensis* [24–27]. Results also suggest the PPF treatment did not affect *Ae*. *albopictus* male and female survivorship or longevity (Fig 5B and 5C and S5 Data). These results are similar to longevity and survivorship assays from field cage trails of the ADAM approach targeting *Ae*. *albopictus*, where little impact on *Ae*. *albopictus* male and female longevity was observed [5]. Indirect transfer of PPF was also not observed to affect the survivorship and longevity of adult *A*. *mellifera* workers (Fig 5D and S5 Data). This observation agrees with earlier findings that also suggested no effect of PPF on the longevity of adult *A*. *mellifera* even at high concentrations [21]. This is in contrast with the findings of Gomes et al. [28], who demonstrated that exposure to PPF through contact with the insect cuticle resulted in increased mortality. Additional experiments are needed to determine the fitness effects of PPF exposure in *A*. *mellifera*, lepidopteran, and other potentially important bee pollinators. However, determining these effects particularly on immature *A*. *mellifera* is complicated by the complex social biology and holometabolous development of *A*. *mellifera* in capped wax cells. Another important bee pollinator species from the genus *Bombus*, which can be maintained as microcolonies in a lab setting, would be a novel system to investigate non-target effects of PPF exposure.

In this study, *Ae*. *albopictus* females were demonstrated to have low levels of PPF contamination in cages with PPF dusted males. Little intraspecific transfer from males to females and/or other indirect transfer was observed in cages housing PPF treated *Ae*. *albopictus* males and untreated females. The observed low level of transfer to females is suggested by the low mortality rates of larvae in bioassays containing female *Ae*. *albopictus* collected from cages with PPF treated males, mass spectrometry, and fluorescent imaging. A low level of mortality was observed in bioassays of females from cages 2 and 3, but little evidence of PPF was observed in mass spectrometry samples collected from cage type 2 (Figs 5 and 6). This is most likely explained by females being contaminated with differing levels of PPF. Specifically, females used in the mass spectrometry analyses were not as contaminated with PPF as those used in the bioassays. However, we would have expected the pooled female mass spectrometry samples to have a higher concentration of PPF than what was observed. Previous studies have suggested males may be directly transferring PPF to females during copulation attempts. We suspect this type of indirect transfer may have been occurring in this study because of the observed low amounts of PPF on *Ae*. *albopictus* females in cage type three housing both PPF treated *Ae*. *albopictus* males and untreated females. However, we cannot rule out that presence of PPF on *Ae*. *albopictus* females was also the result of females visiting PPF contaminated artificial nectar sources. Generally, the presence of females in cages with PPF treated *Ae*. *albopictus* males and *A*. *mellifera* did not impact indirect rates of transfer to *A*. *mellifera*, suggesting PPF treated *Ae*. *albopictus* males are the main contributor for indirectly transferring PPF to *A*. *mellifera* via the artificial nectar source. Additional studies may be required to determine the rate of direct transfer of PPF to females via copulation attempts with PPF treated male mosquitoes.

To our knowledge, no attempts have been made to quantify the amount of PPF on *Aedes* mosquitoes after a dusting treatment for an ADAM approach. While we did not quantify the amount of PPF on an *Ae*. *albopictus* males immediately after dusting, we did quantify the amount of PPF on males at day five at the conclusion of each cage experiment using mass spectrometry (Fig 6 and S6 Data). Data suggests the amount of PPF on individual male *Ae*. *albopictus* is likely to decrease on the insect cuticle over time due to foraging, grooming, mating, contact with substrates, and penetration of PPF into the insect cuticle. [29]. To investigate the persistence of the amount of PPF on treated males, future studies could sample treated male mosquitoes over multiple time periods and the amount of PPF quantified indirectly via bioassays and directly by mass spectrometry.

It remains to be seen that PPF treated male mosquitoes will deliver similar amounts of PPF to that observed in laboratory cages to nectar sources in a more field-like setting. It is also important to note that these experiments were conducted in small laboratory cages where random contact with *A*. *mellifera* and *Ae*. *albopictus* or with the side of contaminated cages could have occurred suggesting that not all PPF observed on *A*. *mellifera* could have originated from visiting a nectar source. However, based on the observation showing *A*. *mellifera* visiting nectar sources without the presence of mosquitoes suggests that the most likely scenario is that honey bees were being contaminated with PPF at the artificial nectars sources. To avoid any space effects, semi-field and field studies would need to be performed in more realistic conditions. Furthermore, these semi-field and field studies will help to determine any additional risks to other non-target insects when using an autodissemination approach. The investigation of PPF transfer from nectar sources to other important insect pollinators (e.g., painted lady butterflies, leafcutter bees, *Bombus* spp., and others) will also be important for determining additional risks in using autodissemination approaches.

The risk of area-wide applications of PPF or other insecticide treatments has not been compared to the risk of using autodissemination approaches. Area-wide spraying of insecticides for insect pests or more specific applications containing an insect growth regulator may be even more harmful to insect pollinator species such as *A*. *mellifera* than the amounts of PPF delivered to the environment via an ADAM approach [9, 30, 31]. With any pesticidal approach, there are risks to non-target organisms and the risk may be acceptable in times of mosquito control need, particularly in association with disease outbreaks. Autodissemination approaches for mosquito control could be a valuable tool to supplement ongoing vector control efforts to reduce arboviral transmission and improve public health even with risks to non-target insects. However, it’s important to understand the level of risk to non-targets and not assume any effects are negligible because PPF is used in small amounts. This study suggests a potential risk to insect pollinators using autodissemination approaches utilizing highly potent insect growth regulators. Additional experiments are needed to investigate whether the observed amounts of PPF exposure via indirect transfer to pollinators in more field-realistic conditions will result in any adverse non-lethal or lethal effects and whether these risks should be considered when designing or utilizing autodissemination approaches for mosquito control.

## Supporting information

S1 Table The proportion of *A*. *mellifera*, *Ae*. *albopictus*, cotton wicks, and filter paper ring samples found to have PPF-fluorescent powder mixture transfer in cage types 1–4. (XLSX)

S1 Data Fecundity data of PPF treated and untreated *D*. *melanogaster* and *Ae*. *albopictus* (Fig 1A & 1B). (XLSX)

S2 Data Bioassay data from experiments examining for dissemination of PPF from treated *Ae*. *albopictus* males to artificial nectar sources (Fig 1F). (XLSX)

S3 Data The number of *A*. *mellifera* found foraging on artificial nectar sources (Fig 1H). (XLSX)

S4 Data Bioassay data from experiments examining for dissemination of PPF from treated *Ae*. *albopictus* males to *A*. *mellifera* (Fig 5A). (XLSX)

S5 Data Survivorship data of *A*. *albopictus* and *A*. *mellifera* in experiments examining for non-target dissemination of PPF (Fig 5B, 5C & 5D). (XLSX)

S6 Data Mass spectrometry data (Fig 6). (XLSX)
